# Novel compound heterozygous ATP6V1B1 mutations in a Chinese child patient with primary distal renal tubular acidosis: a case report

**DOI:** 10.1186/s12882-018-1173-1

**Published:** 2018-12-17

**Authors:** Xiangzhong Zhao, Jingru Lu, Yanxia Gao, Xiaoling Wang, Yanhua Lang, Leping Shao

**Affiliations:** 1 Central Laboratory, The Affiliated Hospital of Qingdao University, 1677 Wutaishan Road, Qingdao, 266555 China; 2 Department of Nephrology, The Affiliated Hospital of Qingdao University, 16 Jiangsu Road, Qingdao, 266003 China; 3 Department of Nephrology, Qingdao Branch of Qilu Hospital of Shandong University, Qingdao, Shandong 266000 People’s Republic of China; 4 Central Laboratory and Department of Nephrolog, the Affiliated Hospital of Qingdao University, 16 Jiangsu Road, Qingdao, 266003 China

**Keywords:** Distal renal tubular acidosis, ATP6V1B1 gene, Sensorineural hearing loss, Enlarged vestibular aqueduct

## Abstract

**Background:**

Distal renal tubular acidosis (dRTA) is a heterogeneous disorder characterized by normal anion gap metabolic acidosis. Autosomal recessive dRTA is usually caused by mutations occurring in ATP6V1B1 and ATP6V0A4 genes,encoding subunits B1 and a4 of apical H^+^-ATPase, respectively. The heterogeneous clinical manifestations of dRTA have been described in different ethnic groups harboring distinct mutations. Most of the reported cases are from Europe and Africa. At present, the prevalence of primary dRTA is still poorly elucidated in Chinese population.

**Case presentation:**

A 2-year and six-month-old female patient was hospitalized because of recurrent hypokalemia, hyperchloremic metabolic acidosis and growth retardation. Laboratory investigations presented a normal anion gap hyperchloremic metabolic acidosis, hypokalemia, and inappropriate alkaline urine. Renal ultrasound indicated bilateral nephrocalcinosis. Bilateral sensorineural hearing loss (SNHL) was confirmed with moderately severe (45 dB) on the left ear and severe (80 dB) on the right ear, which was accompanied with enlarged vestibular aqueduct (EVA) on both sides. According to these findings, a diagnosis of dRTA was made. To identify the pathogenic gene mutation, all coding regions of ATP6V1B1 and ATP6V0A4 gene, including intron-exon boundaries, were analyzed using PCR followed by direct sequence analysis. The splicing variants were verified in peripheral blood leucocytes of the patient by RT-PCR. As a result, two novel heterozygous mutations in ATP6V1B1 were identified in the child. One mutation was a successive 2-nucleotide deletion in exon 2(c.133-134delTG), which caused a marked nonsense mediated mRNA decay. The other was a guanine to adenine substitution of the first nucleotide of intron 8(c.785 + 1 G > A), which led to the exclusion of exon 8. After treatment with sodium citrate, potassium citrateand citric acid, metabolic acidosis and hypokalemia were corrected, but her hearing decreased gradually during the 2 years and had to accept the use of bilateral hearing aids.

**Conclusions:**

We described two novel dRTA associated mutations in ATP6V1B1 identified in a Chinese child patient accompanying with SNHL and EVA. Our study will help to expand the understanding of this rare disease in Chinese population.

## Background

Distal renal tubular acidosis (dRTA) is a rare disease resulting from a failure of the secretion of hydrogen ions in the distal nephron [1–3]. This disorder is characterized by hyperchloremic (normal anion gap) metabolic acidosis, and often accompany with hypokalemia, inappropriately alkaline urine, nephrocalcinosis, and/or nephrolithiasis. It usually has an early age onset and leads to failure to thrive [4–7].

Congenital dRTA can be caused by autosomal dominant or recessive gene defects. So far, three genes (SLC4A1, ATP6V0A4 and ATP6V1B1)responsible for this disease have been identified, each of which encodes the proteins expressed in a-intercalated cells of the collecting duct: basolateral Cl/HCO3 exchanger AE1, B1 and a4 subunits of the apical H^+^-ATPase, respectively [5, 8]. SLC4A1 mutations are mainly responsible for autosomal dominant cases of dRTA. Whereas,ATP6V1B1 and ATP6V0A4 mutations account for recessive dRTA, which often presents complicated genetic heterogeneity especially in the auditory phenotype. Cases bearing ATP6V1B1 mutations usually accompany with early sensorineural hearing loss (SNHL), while ones caused by ATP6V0A4 mutations commonly comorbid with late-onset SNHL or normal hearing [4, 9]. But that’s not exactly true, some cases harboring ATP6V1B1 mutations without SNHL, and a few cases with ATP6V0A4 mutations with early-onset SNHL also have been described [10, 11]. In addition, in our previous report including six Chinese children patient with dRTA, we noticed that enlarged vestibular aqueduct (EVA), a special pathological change of inner ear, was almost concurrent with early onset SNHL regardless of mutations in ATP6V1B1 or in ATP6V0A4 [12]. All these indicated a complexity of audiological phenotype in recessive dRTA patients.

In this paper, we analyzed the causal genes in a Chinese child patient with dRTA, and explored its correlation with audioloical phenotype. This report will expand our understanding of the current prevalence and characteristics of dRTA in China.

## Case presentation

The proband was a 2-year and six-month-old female patient from healthy unrelated parents at full-term normal delivery and with a birth of weight of 3.2 kg. Her perinatal period was unremarkable. At around 4 months of age, she was admitted to the local hospital due to vomiting. At that time, the laboratory finding showed that she suffered from metabolic acidosis and hypokalemia (Table 1). She accepted the supplementary treatment of potassium chloride and sodium bicarbonate for a short time and then therapy was discontinued. At about 2.5 years old, she was hospitalized in our renal unit because of recurrent hypokalemia, hyperchloremic metabolic acidosis and growth retardation. Physical examinations on admission showed height (84.0 cm, <3rd percentile) and weight (10.1 kg, <3rd percentile) were lower than normal. Clinical features and biochemical data revealed that the patient presented hypokalemia (2.7 mmol/l, normal 3.5–5.5 mmol/l),hyperchloremic (115 mmol/l, normal 99-110 mmol/l), metabolic acidosis (pH 7.28, normal7.35–7.45) and paradoxical alkali urine (Urinary pH > 6.0 while CO_2_CP < 18 mmol/l) (Table 1). Thus, the clinical and biochemical features of this patient suggested a diagnosis of dRTA.

**Table 1:** Clinical features and biochemical data of the female child at the age of symptom-onset, diagnosis and the last follow-up visit

Items	Age of onset	Age of diagnosis	Current age	Normal range
Age (yrs)	0.33	2.5	4.5	
Manifestation	Vomiting	Growth retardation	Growth normal	
Height (cm)	60.0 (15th percentile)^a^	84.0 (< 3rd percentile)^a^	110.0 (+1SD)^a^	
Weight (kg)	5.4 (<15th percentile)^a^	10.1 (<3rd percentile)^a^	19.8 (+1SD)^a^	
Blood pH	7.20	7.28	7.40	7.35–7.45
Serum K^+^ (mmol/l)	2.2	2.7	4.4	3.5–5.5
Serum Na^+^ (mmol/l)	140	138	140	135–145
Serum Cl^−^ (mmol/l)	112	115	105	99–110
Serum CO_2_CP (mmol/l)	18.0	16.7	24.5	22–28
Serum ionized Ca^2+^ (mmol/l)	1.28	1.33	1.20	1.10–1.30
Serum Cr (μmol /l)	25 (16–26)^b^	29 (17.7–88.4)^b^	40 (17.7–88.4)^b^	
GFR (ml/min/1.73m^2^)^c^	87 (39–114)^b^	105 (89–165)^b^	100 (89–165)^b^	
Nephrocalcinosis	NA	Yes	Yes	Negative
Urinary pH	7.5	7.0	7.0	< 5.5
Urinary Ca/Cr ratio (mg/mg)	NA	0.63 (0.02–0.50)^b^	0.35 (0.02–0.41)^b^	
Proteinuria	Negative	Negative	Negative	Negative

*K* potassium, *Na* sodium, *Cl* chloridion, *CO*_*2*_*CP* Carbon Dioxide Combining Power, *Ca* calcium, *Cr* creatinine, *GFR* glomerular filtration rate, ^a^percentiles/standard deviation (SD) scores for height or weight,^b^Figures in the brackets indicate normal ranges of the corresponding age, ^c^GFR was estimated by Schwartz equation, *NA* Not available.

**Fig. 1:**
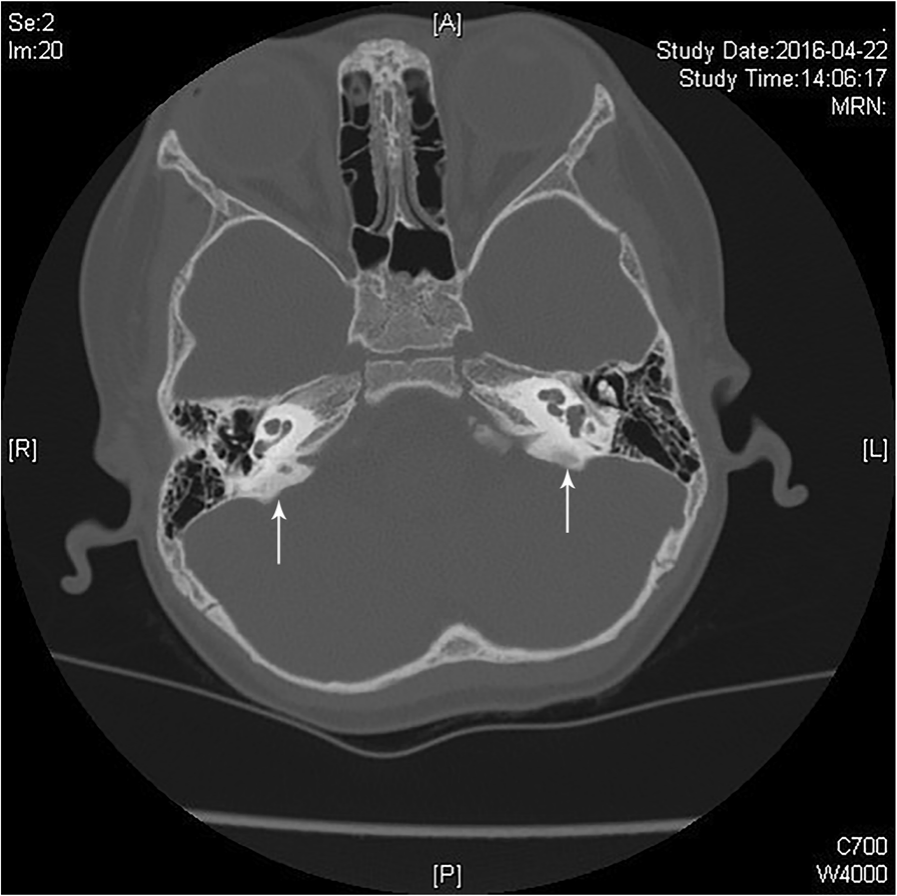
High-resolution computed tomography indicated bilateral enlargement of the vestibular aqueduct

To make a definite diagnosis, renal ultrasound and audiological assessment were performed. Renal ultrasound indicated bilateral nephrocalcinosis. Automated auditory brainstem response (AABR) test revealed that bilateral sensorineural hearing loss, with moderately severe (45 dB) on the left ear and severe (80 dB) on the right ear, which was accompanied with EVA on both sides determined by high-resolution computed tomography (HR-CT) (Fig. 1).

**Fig. 2:**
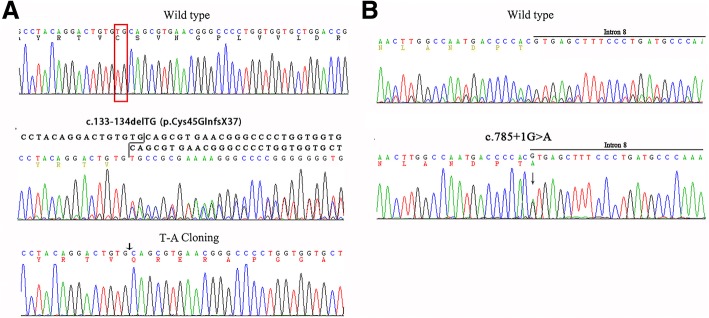
Two novel ATP6V1B1 mutations identified in a Chinese patient with dRTA. **a** Partial nucleotide sequence of the wild type and the successive 2-nucleotide deletion in exon 2(c.133-134delTG). The arrow indicated the position of deleted TG in exon 2. **b** The guanine to adenine substitution of the first nucleotide of intron 8(c.785 + 1 G > A). The arrow indicated the position of G > A mutation in intron 8

To identify the pathogenic gene mutation, Genomic DNA was extracted from the peripheral blood of the patient and her parents using Blood genome DNA Extraction kit (TaKaRa, Japan). Both ATP6V1B1 and ATP6V0A4 genes were preferentially screened in this study. If inconclusive (no mutation or only one was identified in either gene) then SLC4A1 gene should be analyzed for further verification. Direct sequencing analysis was employed to screen both of ATP6V1B1 and ATP6V0A4 genes, and two novel mutations were identified in ATP6V1B1. One mutation was a successive 2-nucleotide deletion in exon 2(c.133-134delTG)(Fig. 2), which resulted in a frame shift mutation (p.Cys45Glnfs*37) and was expected to produce a truncated protein. The other mutation was a guanine to adenine substitution of the first nucleotide within the intron 8(c.785 + 1 G > A)(Fig. 2). No mutation was found in ATP6V0A4 and we did not perform the SLC4A1 gene analysis since the causal mutations have been found.

For the mutation in the first nucleotide of intron 8(c.785 + 1 G > A), which just located in the 5′-splice donor site, splicing prediction programs presumed this mutation cause the disability of donor site and skipping exon 8with the on-line software BDGP (Score decreases from 0.92 to 0), NetGene2 (Confidence decreases from 0.88 to 0) and Spliceview (Score decreases from 85.6 to 0), respectively.

**Fig. 3:**
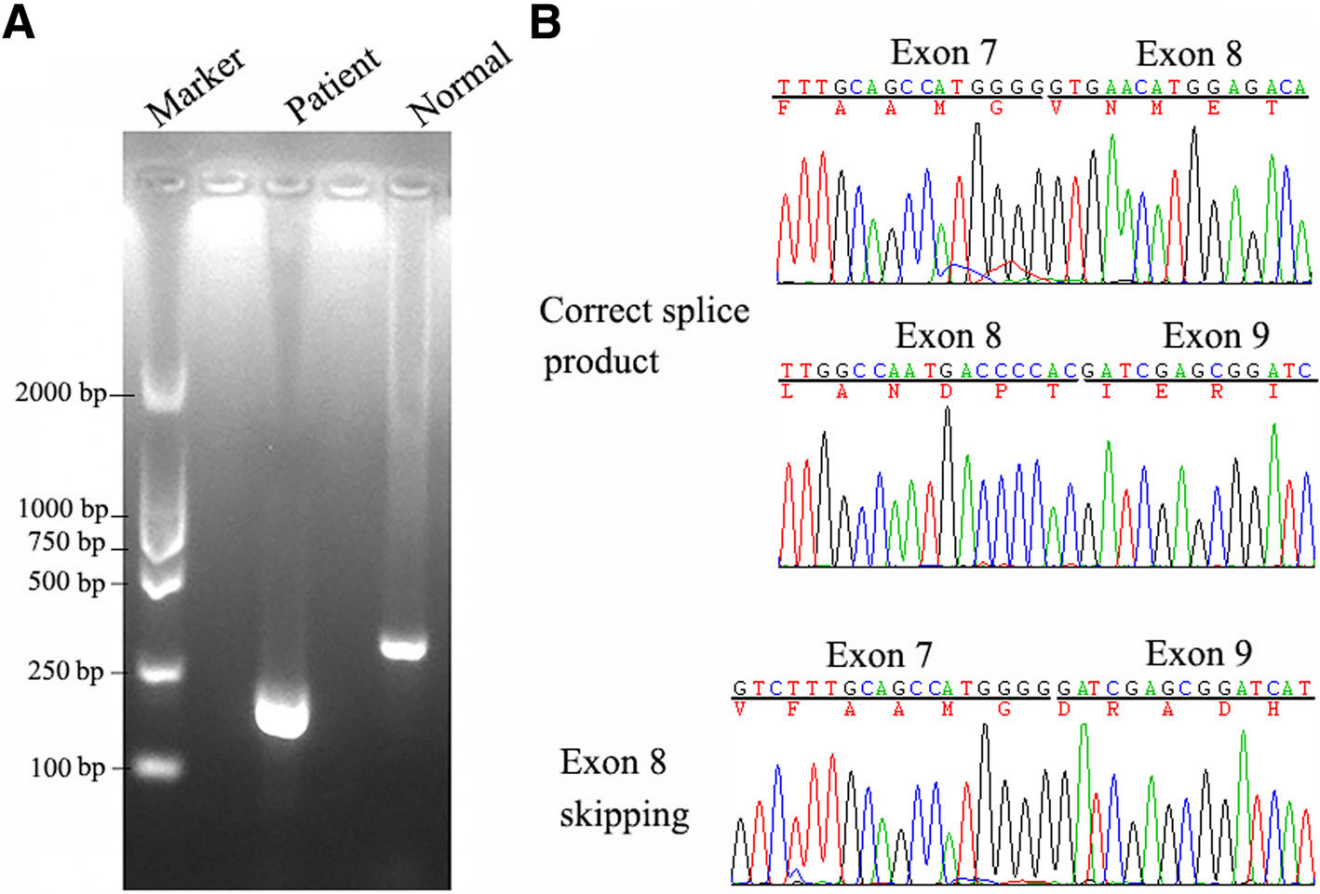
The verification of exon 8 skipping in the peripheral white cells of the patient. The cDNA segment containing exon 7, 8 and 9 of ATP6V1B1 were amplified by nested PCR as above-described. **a** Electrophoresis of the PCR products from normal control or the patient. **b** Sequencing chromatogram of the PCR products from normal person (correct splice product) or the patient (exon 8 skipping)

To verify this mutation really led to exon 8 skipping in vivo, the cDNA from the peripheral blood of the patient was amplified by nested PCR with primers spanning exon 7 to exon 9 (Table 2). By direct PCR products sequencing, the exon 8-excluded transcript was visualized, while the normal was not (Fig. 3). Of note, the absence of RT-PCR product corresponding to the allele harboring c.133-134delTG from this patient might suggest a marked nonsense mediated mRNA decay (NMD). The parents of this patient gave their informed consent. The study protocol was approved by the Ethics Committee of the affiliated hospital of Qingdao University.

**Table 2:** Nested PCR primers for analysis ATP6V1B1 exon 8 skipping

name	Forward primer (5′–3′)	Reverse primer (5′–3′)	Product (bp)
Exon8-1P	ATCCTACGAACTCCGGTGTC	TATCGTCGTTGGGCATGGTG	730 bp
Exon8-2P	GAGATGATTCAGACGGGCAT	CACCTCCTCTCTAGCAGCAG	450 bp
Exon8-3P	ATGAGATTGCCGCTCAGAT	GCATAGGAACTCATGTCCGT	313 bp

**Fig. 4:**
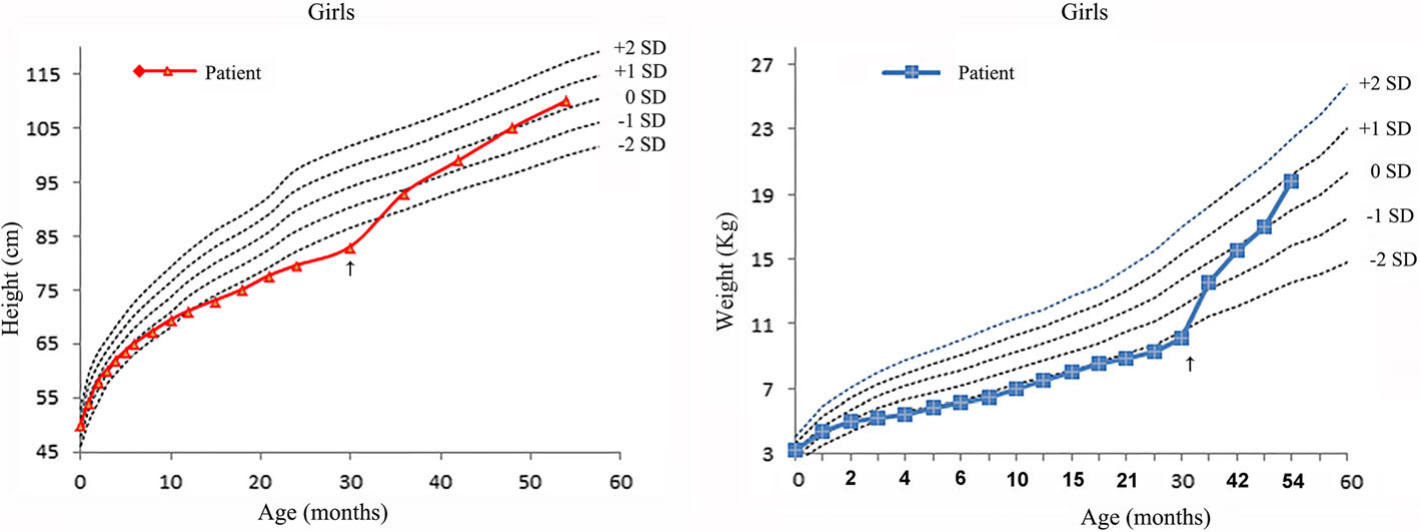
Growth curve of the patient

A systematic treatment was performed by administration of sodium citrate (0.7 mmol/kg/day), potassium citrate (0.65 mmol/kg/day), and citric acid (0.62 mmol/kg/day) to correct metabolic acidosis and hypokalemia in this patient, and her normal growth was also restored in about 2 years (Fig. 4). However, during the follow-up period, from the age of 2.5 to the age of 4.5 years, her hearing decreased gradually with fluctuating exacerbation which was associated with common cold infections. Finally, she had to accept the use of bilateral hearing aids.

## Discussion and conclusions

Mutations in ATP6V1B1 and ATP6V0A4 gene are the main cause responsible for recessive dRTA. To date, more than 30 ATP6V1B1 mutations and 40 ATP6V0A4 mutations have been described [1, 13, 14]. However, only few sporadic cases have been reported in China so far [15, 16]. In our previous study, we reported six Chinese child patients with dRTA and explored the correlation of the phenotype, especially in the aspects of audiological characteristics and the genotype of ATP6V1B1 and ATP6V0A4 mutations [12].

Due to both of ATP6V1B1 and ATP6V0A4 having a relative high expression in human inner ear besides kidney, recessive dRTA patients usually accompany with hearing impairment [17–20]. In our previous report, we assessed the audiology phenotype (SNHL and EVA) and genotype of ATP6V1B1 and ATP6V0A4 mutations in six Chinese children, our results support that EVA concurrent with early onset SNHL cases no matter harboring mutation in ATP6V1B1 or ATP6V0A4.Thus, EVA may be another important feature for phenotype severity [12]. In the present study, we also observe the similar association of EVA, SNHL and ATP6V1B1 mutation in the patient.

Regarding ATP6V1B1 mutations, most of them are disease-related missense or nonsense. Other mutations described are small insertions, small deletions and splicing mutations [21]. The pathogenic mechanisms of ATP6V1B1 gene mutations have been well understood in recent years. Failure of V-ATPase assembly was the common underlying mechanism of B1 subunit-associated human disease [22]. Almost known missense mutations such as L81P, R124W, M174R, T275P, G316E, were verified in their effects on H-ATPase assembly or trafficking in culture cells or yeasts ([23, 24]. In the present report, we found a successive 2-nucleotide deletion in exon 2(c.133–134 delTG), which resulted in a frame-shift mutation (p.Cys45Glnfs*37) and was previously predicted to produce a very short truncated protein, which lack C-terminal region which was critical for inter-subunits assembly of H^+^-ATPase [24]. However, this mutation was eventually proved to lead to a marked NMD by RNA analysis. Another mutation is a guanine to adenine substitution of the first nucleotide of intron 8(c.785 + 1 G > A), which cause the exclusion of the whole exon 8. This mutation is very similar to our previously reported mutation C786–1 G > C which was supposed to lead to the exclusion of exon 8, resulting in a frame shift from codon 230 and premature termination at position 244 and a truncated protein [12].

In conclusion, we identified two novel compound heterozygous ATP6V1B1 mutations in a Chinese patient with dRTA, and confirmed their functional consequences by in vivo analysis of RNA from white blood cells for the first time. In addition, we further confirmed the association of audiological phenotype and genotype in this patient, which will help to expand our understanding for this disease in China.

## Abbreviations

AABR: Automated auditory brainstem response
dRTA: Distal renal tubular acidosis
EVA: Enlarged vestibular aqueduct
HR-CT: High-resolution computed tomography
SNHL: Early sensorineural hearing loss
